# Clinical sequelae among individuals with pauci-symptomatic or asymptomatic Ebola virus infection and unrecognised Ebola virus disease in Liberia: a longitudinal cohort study

**DOI:** 10.1016/S1473-3099(22)00127-X

**Published:** 2022-05-16

**Authors:** J Daniel Kelly, Collin Van Ryn, Moses Badio, Tamba Fayiah, Kumblytee Johnson, Dehkontee Gayedyu-Dennis, Sheri D Weiser, Travis C Porco, Jeffery N Martin, Michael C Sneller, George W Rutherford, Cavan Reilly, Mosoka P Fallah, J Soka Moses

**Affiliations:** Department of Epidemiology and Biostatistics (J D Kelly MD, M Badio MS, Prof T C Porco PhD, Prof J N Martin MD, Prof G W Rutherford MD), Institute for Global Health Sciences (J D Kelly, Prof G W Rutherford), F I Proctor Foundation (J D Kelly, Prof T C Porco) and Division of HIV, Infectious Disease, and Global Medicine (Prof S D Weiser MD), University of California, San Francisco, CA, USA; Partnership for Research on Vaccines and Infectious Diseases in Liberia (PREVAIL), Monrovia, Liberia (J D Kelly, M Badio, T Fayiah BSc, K Johnson MD, D Gayedyu-Dennis MD, Prof C Reilly PhD, M P Fallah PhD, J S Moses MD); Division of Biostatistics, University of Minnesota, Minneapolis, MN, USA (C Van Ryn MS, Prof C Reilly); Laboratory of Immunoregulation, National Institute of Allergy and Infectious Diseases, Bethesda, MD, USA (Prof M C Sneller MD); A M Dogliotti College of Medicine, University of Liberia, Monrovia, Liberia (M P Fallah)

## Abstract

**Background:**

Whether or not individuals with pauci-symptomatic or asymptomatic Ebola virus infection and unrecognised Ebola virus disease develop clinical sequelae is unknown. We assessed current symptoms and physical examination findings among individuals with pauci-symptomatic or asymptomatic infection and unrecognised Ebola virus disease compared with Ebola virus disease survivors and uninfected contacts.

**Methods:**

Between June 17, 2015, and June 30, 2017, we studied a cohort of Ebola virus disease survivors and their contacts in Liberia. Surveys, current symptoms and physical examination findings, and serology were used to characterise disease status of reported Ebola virus disease, unrecognised Ebola virus disease, pauci-symptomatic or asymptomatic Ebola virus infection, or no infection. We pre-specified findings known to be differentially prevalent among Ebola virus disease survivors versus their contacts (urinary frequency, headache, fatigue, muscle pain, memory loss, joint pain, neurological findings, chest findings, muscle findings, joint findings, abdominal findings, and uveitis). We estimated the prevalence and incidence of selected clinical findings by disease status.

**Findings:**

Our analytical cohort included 991 reported Ebola virus disease survivors and 2688 close contacts. The median time from acute Ebola virus disease onset to baseline was 317 days (IQR 271–366). Of 222 seropositive contacts, 115 had pauci-symptomatic or asymptomatic Ebola virus infection and 107 had unrecognised Ebola virus disease. At baseline, prevalent findings of joint pain, memory loss, muscle pain, and fatigue were lowest among those with pauci-symptomatic or asymptomatic infection or no infection, higher among contacts with unrecognised Ebola virus disease, and highest in reported survivors of Ebola virus disease. Joint pain was the most prevalent finding, and was reported in 434 (18%) of 2466 individuals with no infection, 14 (12%) of 115 with pauci-symptomatic or asymptomatic infection, 31 (29%) of 107 with unrecognised Ebola virus disease, and 476 (48%) of 991 with reported Ebola virus disease. In adjusted analyses, this pattern remained for joint pain and memory loss. Survivors had an increased odds of joint pain compared with unrecognised Ebola virus disease contacts (adjusted odds ratio [OR] 2·13, 95% CI 1·34–3·39); unrecognised Ebola virus disease contacts had an increased odds of joint pain compared with those with pauci-symptomatic or asymptomatic infection and uninfected contacts (adjusted OR 1·89, 95% CI 1·21–2·97). The adjusted odds of memory loss was more than four-times higher among survivors than among unrecognised Ebola virus disease contacts (adjusted OR 4·47, 95% CI 2·41–8·30) and two-times higher among unrecognised Ebola virus disease contacts than in those with pauci-symptomatic or asymptomatic infection and uninfected contacts (adjusted OR 2·05, 95% CI 1·10–3·84). By 12 months, prevalent findings had decreased in the three infected groups.

**Interpretation:**

Our findings provide evidence of post-Ebola virus disease clinical sequelae among contacts with unrecognised Ebola virus disease but not in people with pauci-symptomatic or asymptomatic Ebola virus infection.

**Funding:**

National Cancer Institute and National Institute of Allergy and Infectious Diseases of the National Institutes of Health.

## Introduction

The 2013–16 Ebola virus disease outbreak in western Africa resulted in more than 28 000 reported cases and prompted intensive study of survivors to understand the clinical complications of their acute illness and create comprehensive care programmes.^1–4^ Survivors of Ebola virus disease experience a wide spectrum of clinical sequelae, ranging from uveitis and memory loss to headache and muscle pain.^5–9^ Longer-term study of survivors found that most of these conditions declined in prevalence over a 12-month follow-up period (1–2 years after Ebola virus disease onset),^7^ but a substantial proportion of post-Ebola virus disease sequelae persisted for as long as 2·5–4 years after Ebola treatment centre discharge.^10,11^

Individuals with suspected Ebola virus disease are typically categorised as survivors if they were diagnosed as PCR-positive for Ebola virus, discharged alive from a health-care facility or Ebola treatment facility, and listed in an Ebola virus disease registry.^12^ In the post-Ebola virus disease period, survivors have been the focus of international research and programmatic efforts, with provision of clinical, psychosocial, and economic support from governmental and non-governmental programmes.^13–15^ A few studies have also shown a substantial burden of pauci-symptomatic or asymptomatic Ebola virus infection and unrecognised Ebola virus disease.^16–19^ A large proportion of the latter group never presented to an Ebola treatment facility while infected and were therefore not diagnosed with Ebola virus disease or recorded survivors of the illness. In addition to those with pauci-symptomatic or asymptomatic infection, unrecognised Ebola virus disease survivors might have also had, on average, less severe acute disease than those diagnosed and treated at Ebola treatment facilities.^7^ Both groups of individuals—those with pauci-symptomatic or asymptomatic infection and those with unrecognised Ebola virus disease—might have developed post-infectious clinical sequelae.

Little evidence exists linking acute Ebola virus disease illness and its severity to clinical sequelae; however, studies have shown that particular symptoms (eg, haemorrhage, and neurological and abdominal symptoms) or higher viraemia, or both during acute Ebola virus disease correlated with subsequent clinical sequelae.^10,20,21^ These studies raised the question of whether a dose–response relationship exists between the severity of acute Ebola virus disease and subsequent clinical sequelae. However, the small size of previous Ebola virus disease outbreaks and their occurrence mostly in settings with under-resourced health systems prevented systematic study of this hypothesis in individuals with pauci-symptomatic or asymptomatic infection and unrecognised Ebola virus disease.

During the 2013–16 Ebola virus disease outbreak in Liberia, we identified a large cohort of Ebola virus disease survivors and contacts, inclusive of individuals with pauci-symptomatic or asymptomatic infection and unrecognised Ebola virus disease who can be assessed for evidence of clinical sequelae. Since individuals with pauci-symptomatic or asymptomatic infection and unrecognised Ebola virus disease probably had less severe acute disease than reported survivors, we hypothesised that individuals with less severe acute disease experience post-Ebola virus disease clinical sequelae to a lesser extent than reported Ebola virus disease survivors, and that a viral load-dependent relationship exists between the severity of acute illness and clinical sequelae.

## Methods

### Study design and participants

We used data from a longitudinal cohort study (PREVAIL III; NCT02431923) of Ebola virus disease survivors and close contacts in Liberia, implemented through a partnership between the Ministry of Health in Liberia and the National Institute of Allergy and Infectious Diseases (NIAID). A primary objective of the PREVAIL III study is to determine the sequelae of Ebola virus infection. Enrolment occurred at three research sites in Liberia (John F Kennedy Medical Center and Duport Road Clinic in Monrovia, and C H Rennie Hospital in Kakata) from June 17, 2015, to June 30, 2017. The methods and findings of the primary study have previously been published.^7^ In brief, PREVAIL III enrolled Ebola virus disease survivors who were listed in the Liberian Ministry of Health Registry and had a documented diagnosis of the disease. These survivors listed their close contacts, who were then eligible to be enrolled. A close contact was an individual selected by an Ebola virus disease survivor as someone with whom the survivor had contact during acute Ebola virus disease or with whom they had sexual contact following acute illness. Survivors and close contacts underwent study visits every 6 months that included a symptom checklist, physical examination, and collection of blood. A subset of participants was referred for a separate eye examination.^7^

The study protocol was approved by the National Research Ethics Board of Liberia, the University of California, San Francisco Institutional Review Board, and the NIAID Institutional Review Board at the US National Institutes of Health. Written informed consent was obtained from all participants.

### Measurements

Serum samples were analysed at the Liberian Institute for Biomedical Research, as previously described^7^ for anti-glycoprotein Ebola virus antibody concentrations using the Filovirus Animal Non-Clinical Group assay. A cutoff of 548 enzyme-linked immunosorbent assay units (EU) per mL was used to determine seropositivity with 94·4% sensitivity and 96·7% specificity.^7^

The explanatory variable included four groups: Ebola virus disease survivors (seropositive); contacts with unrecognised Ebola virus disease (seropositive); contacts with pauci-symptomatic or asymptomatic Ebola virus infection (seropositive); and uninfected contacts (seronegative). We assumed that individuals who were seropositive had an Ebola virus infection following exposure; we then used the serostatus and self-reported post-exposure symptoms of each participant to determine group membership. Self-reported symptoms were determined with the following question: “Did you develop any of the following symptoms within 21 days of the survivor’s Ebola event?” The checklist of self-reported symptoms included 16 items from the WHO Ebola virus disease case definition,^22^ with responses selected as a binary (yes or no). We compiled these responses and classified each contact participant as either asymptomatic or symptomatic. Participants who were seropositive and asymptomatic and responded no to all 16 questions were classified as having had a pauci-symptomatic or asymptomatic infection. We selected pauci symptomatic or asymptomatic as the description of individuals who reported being asymptomatic because of the potential for mild symptoms with recall error. Contact participants who were seropositive and symptomatic were defined as having had unrecognised Ebola virus disease.

Severity of acute Ebola virus disease illness was ranked (less severe first) by the following categories of disease: no infection; pauci-symptomatic or asymptomatic infection; unrecognised Ebola virus disease; and patients with reported and confirmed Ebola virus disease who survived. The individuals with unrecognised Ebola virus disease did not receive testing or medical care during their illness, were more likely to remain at home through the duration of their infection, and reported, on average, fewer symptoms than reported Ebola virus disease survivors during their post-exposure period.^7^

Outcomes were defined as current symptoms and physical examination findings reported at each study visit. The current symptoms and physical examination findings reported in this analysis were limited to those found to be statistically significantly more or less prevalent between survivors and close contacts at baseline (p<0·0001) in the parent study by Sneller and colleagues.^7^ Symptoms were urinary frequency, headache, fatigue, muscle pain, memory loss, and joint pain. Physical examination findings were neurological findings, chest (respiratory) findings, muscle findings, joint findings, abdominal findings, and uveitis.

### Statistical analysis

Categorical baseline factors were compared between groups using χ2 tests, age at enrolment was compared between groups using one-way ANOVA, and baseline antibody concentrations were compared between survivors and close contact groups using linear regression models with generalised estimating equations (GEE) that adjusted for relationships between survivors and close contacts with an independent correlation. Comparisons of the prevalence of self-reported symptoms and abnormal findings on physical examination at the baseline and 12-month study visits were analysed using GEE logistic regression models. All models were adjusted for age at PREVAIL III enrolment, sex, and enrolment site, except for models comparing uveitis at the 12-month visit, which was only adjusted for age and sex. These GEE models adjusted for potential correlation of outcomes within groups of survivors and associated close contacts.

We did time-to-event analyses for symptoms, physical examination findings, hospitalisation, and death within 1 year of enrolment. Incidence rates per 1000 person-years of symptoms and physical examination findings are reported for individuals without symptoms or findings at baseline. Time-to-event for symptoms and hospitalisation was calculated as the number of days from enrolment to the date of the 6-month or 12-month follow-up visit: either the first at which a symptom or hospitalisation was reported, or, if neither were reported, the last of these follow-up visits to occur. Survival time was calculated as the number of days from enrolment to death, follow-up discontinuation date, or the 1-year anniversary of enrolment, whichever occurred first. The occurrence of symptoms, findings on physical examination, hospitalisation, and death within 1 year of enrolment were compared between groups using Cox proportional hazard models, which adjusted for age, sex, and enrolment site, except the model for uveitis which only adjusted for age and sex. GEE were used to adjust for potential correlation of outcomes within groups of survivors and associated close contacts. We estimated hazard ratios from these Cox models. All analyses were done using R version 3.3.2, and p values less than 0·05 were considered statistically significant.

### Role of the funding source

The sponsor of the study had no role in study design, data collection, data analysis, data interpretation, or writing of the report.

## Results

**Figure 1:**
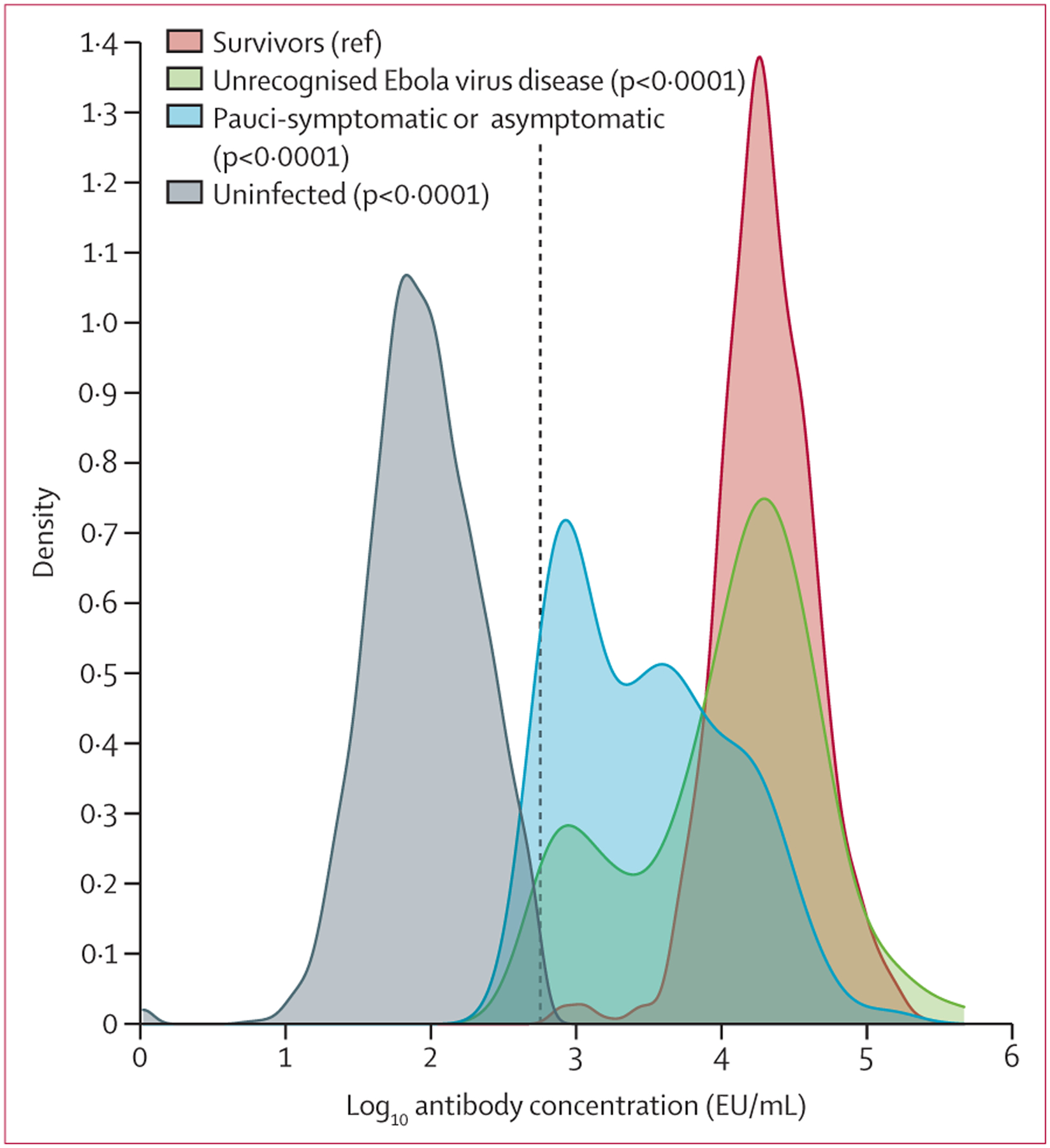
Antibody concentrations by group and p values for tests comparing concentrations in the close contact groups with Ebola virus disease survivors Concentrations were compared using generalised estimating equation linear regression models that adjusted for relationships between survivors and close contacts. The dashed vertical line indicates the cutoff for seropositivity.

Among 3679 participants, 991 (27%) were reported Ebola virus disease survivors, 107 (3%) were contacts with unrecognised Ebola virus disease, 115 (3%) were contacts with pauci-symptomatic or asymptomatic infection, and 2466 (67%) were uninfected contacts. The median time from acute Ebola virus disease onset to baseline was 317 days (IQR 271–366). Among all 3679 participants, 2048 (56%) were female, and the median age was 25 years (IQR 15–36). The baseline characteristics of the analysis cohort are shown in table 1. Ebola virus disease survivors had higher median antibody concentrations than other groups (figure 1). At month 12, 412 (11%) of 3679 participants had been lost to follow-up.

**Figure 2:**
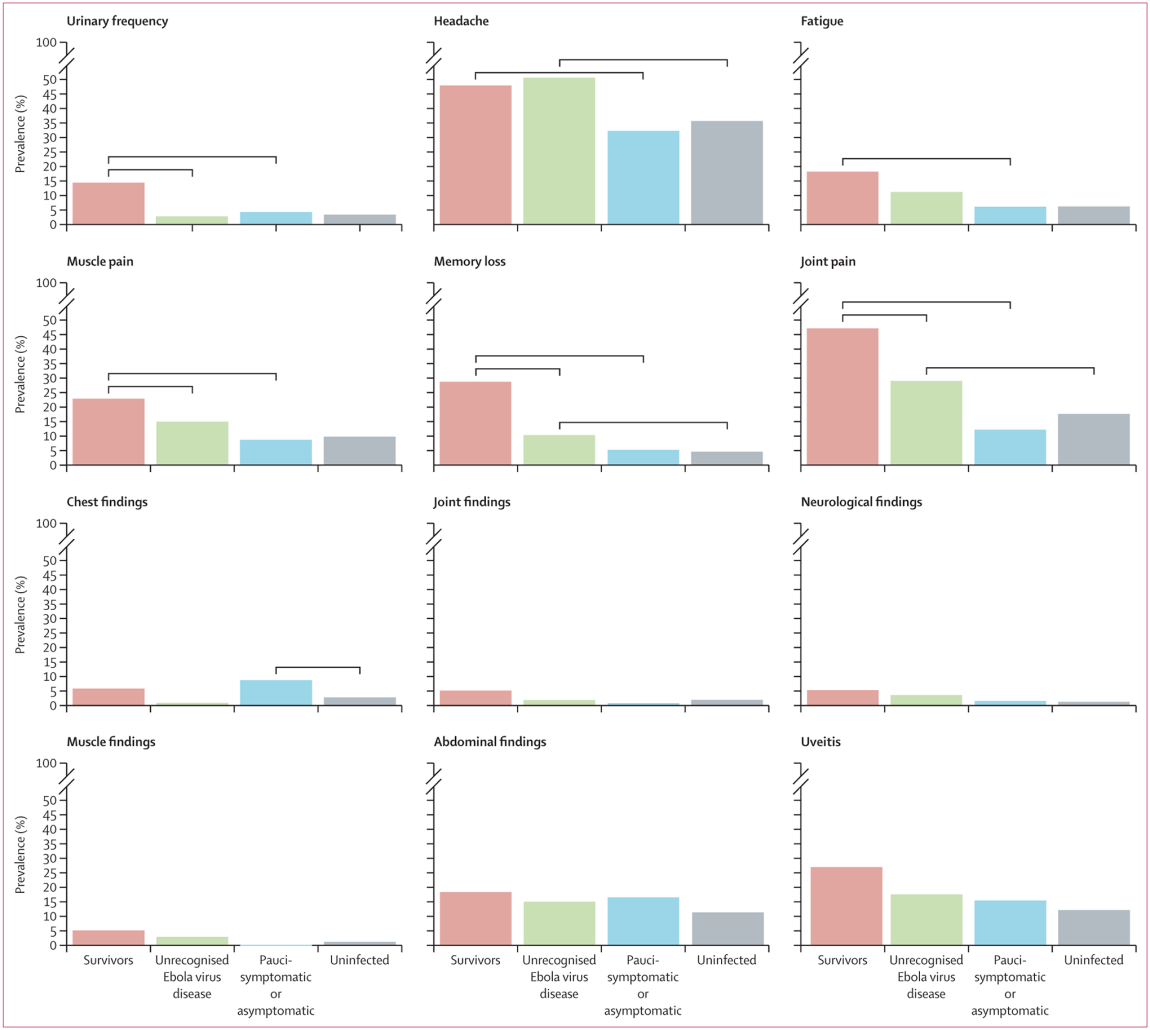
Prevalence of selected symptoms and findings on physical examination at baseline Statistically significant comparisons (p<0·05) are indicated by horizontal brackets. Survivors were compared with the unrecognised Ebola virus disease and pauci-symptomatic or asymptomatic groups, and the unrecognised Ebola virus disease and pauci-symptomatic or asymptomatic groups were compared to the uninfected group. Comparisons were made using logistic regression models that adjusted for age, sex, and enrolment site (except the model for uveitis, which adjusted for age and sex only) and used generalised estimating equations to adjust for relationships between survivors and close contacts.

We determined the prevalence of selected symptoms and physical examination findings and created a graphical representation for each group at study baseline (figure 2). Table 2 shows the prevalence of the selected symptoms and physical examination findings in each group at baseline and month 12.

At baseline, we observed a stepped increase in prevalent findings across groups. Prevalent findings of joint pain, memory loss, muscle pain, and fatigue were lowest among those with pauci-symptomatic or asymptomatic or no infection, higher among contacts with unrecognised Ebola virus disease, and highest among Ebola virus disease survivors. The trend was clearest in the report of joint pain (434 [18%] of 2466 uninfected contacts *vs* 14 [12%] of 115 pauci-symptomatic or asymptomatic contacts *vs* 31 [29%] of 107 contacts with unrecognised Ebola virus disease *vs* 467 [47%] of 991 Ebola virus disease survivors), memory loss (113 [5%] *vs* 6 [5%] *vs* 11 [10%] *vs* 284 [29%]), muscle pain (242 [10%] *vs* 10 [9%] *vs* 16 [15%] *vs* 227 [23%]), and fatigue (152 [6%] *vs* 7 [6%] *vs* 12 [11%] *vs* 180 [18%]; table 2).

In adjusted analyses, this pattern remained for joint pain and memory loss at baseline. Survivors had an increased likelihood of joint pain compared with contacts with unrecognised Ebola virus disease (adjusted odds ratio [OR] 2·13, 95% CI 1·34–3·39), while contacts with unrecognised Ebola virus disease had a similarly increased odds of joint pain compared with contacts with pauci-symptomatic or asymptomatic infection and uninfected contacts (adjusted OR 1·89, 95% CI 1·21–2·97; table 3). The largest magnitude of association that followed this pattern was for memory loss. Compared with contacts with unrecognised Ebola virus disease, survivors had a higher adjusted odds of memory loss, (adjusted OR 4·47, 95% CI 2·41–8·30), while contacts with unrecognised Ebola virus disease had a higher adjusted odds of memory loss compared with pauci-symptomatic or asymptomatic contacts and uninfected contacts (2·05, 1·10–3·84; table 3).

Other patterns emerged from the results but were not consistently observed across multiple outcomes. For three symptoms (urinary frequency, muscle pain, and uveitis), each finding was most prevalent in Ebola virus disease survivors and observed at similar prevalence among the other three groups. For example, urinary frequency was observed in 143 (14%) of 991 reported Ebola virus disease survivors but only in three (3%) of 107 unrecognised Ebola virus disease survivors, five (4%) of 115 pauci-symptomatic or asymptomatic contacts, and 83 (3%) of 2466 uninfected contacts. For one symptom (headache), the finding had similarly high prevalence among reported and unrecognised Ebola virus disease survivors but was equally low among pauci-symptomatic or asymptomatic and uninfected contacts. In adjusted analyses, these patterns remained for urinary frequency, muscle pain, and headache, but not uveitis (table 3).

From baseline to the 12-month visit, the selected symptoms and clinical findings generally decreased in prevalence (table 2). As a result, most of the statistical associations present at baseline were no longer observed at 12 months (table 3).

During the same 12-month study period, participants reported the new occurrence of selected symptoms and clinical findings that were not reported at baseline. These incident findings occurred among fewer participants than prevalent findings (tables 2, 4). We compared these incident findings among groups to assess for potential patterns. Three incident findings (headache, memory loss, and chest findings) were more likely to occur in Ebola virus disease survivors or contacts with unrecognised Ebola virus disease than among contacts with pauci-symptomatic or asymptomatic infection or uninfected contacts. For headache, the adjusted hazard ratio (HR) in survivors versus contacts with pauci-symptomatic or asymptomatic infection was 2·33 (95% CI 1·29–4·22). Compared with uninfected contacts, contacts with unrecognised Ebola virus disease had a higher likelihood of memory loss (adjusted HR 9·61, 95% CI 1·86–49·80) and of chest findings (2·62, 1·09–6·29). We did not find any differences in the rates of hospitalisation or mortality among the groups (table 4).

## Discussion

In this longitudinal cohort in Liberia following the 2013–16 Ebola outbreak, we found evidence of post-Ebola virus disease clinical sequelae in contacts with unrecognised Ebola virus disease but not in contacts with pauci-symptomatic or asymptomatic Ebola virus infection. Previous cohort studies of Ebola virus disease survivors were smaller in size, so even if they had identified a group of contacts with pauci-symptomatic or asymptomatic infection and unrecognised Ebola virus disease, they were underpowered, did not use a control group, and were unable to reliably identify the presence or absence of post-Ebola virus disease symptoms and examination findings considered to be clinical sequelae.^23,24^ Our findings were consistent for multiple symptoms (memory loss, headache, and joint pain), which adds strength to the evidence that post-Ebola virus disease sequelae occur among unrecognised Ebola virus disease contacts. Once contacts with post-exposure Ebola virus disease symptoms are identified as seropositive in future outbreaks, the Ebola virus disease response community should screen this group for post-Ebola virus disease clinical sequelae and offer clinical care and support services.

We found patterns that more severe acute illness has the potential to cause specific types of post-Ebola virus disease clinical sequelae. In particular, memory loss, joint pain, headache, and urinary frequency were observed at higher prevalence across groups (with the prevalence much higher in Ebola virus disease survivors than in those with unrecognised Ebola virus disease, who in turn had a higher or similar prevalence to those with no or pauci-symptomatic or asymptomatic infection). Other studies have shown that specific features of acute illness (symptoms associated with more severe illness or degree of viraemia) can lead to post-Ebola virus disease sequelae, including uveitis and joint pain,^10,21^ so our findings support this growing body of evidence. We also extend the evidence for a viral load-dependent association between acute illness and clinical sequelae,^21^ by showing its occurrence across the spectrum of clinical manifestations, particularly in individuals with unrecognised Ebola virus disease (a group identified as having fewer symptoms than reported Ebola virus disease survivors during acute illness^7^). Our proof-of-concept study offers insight into the types of post-Ebola virus disease clinical sequelae potentially observed in the clinical setting; however, we need natural history studies that prospectively enrol individuals with asymptomatic infection and mild illness during the acute phase and follow them into the convalescence phase in order to confirm our findings.

We acknowledge several limitations to our study. We do not have data (biological, clinical, social, or psychological) from the pre-enrolment period, including acute illness, so there is potential unmeasured confounding. Given the absence of within-participant measurements starting from the acute illness, we cannot definitively consider the reported current symptoms or physical examination findings as post-Ebola virus disease clinical sequelae. Enrolment started nearly 1 year after survivors were discharged from an Ebola treatment facility, and those individuals who were sicker may have been more likely to participate, which might be a source of selection bias. Our classification of contacts as seropositive or seronegative cannot be used to confirm infection because of potential cross-reactivity and measurement error, but the test performance characteristics of the immunoassay used in this study are highly accurate,^7^ robust over time,^25,26^ and have been used in several other high-impact studies.^7,27^ In terms of external validity, Ebola virus vaccines were introduced at the end of this outbreak (in October, 2015),^28^ so our findings represent the potential for post-Ebola virus disease clinical sequelae in an unvaccinated population. Our study also had several strengths, including the use of a control group to demonstrate between-group differences and sufficient power to draw reliable conclusions across most groups. However, we did not have sufficient power to compare unrecognised Ebola virus contacts against contacts with pauci-symptomatic or asymptomatic infection, even though to our knowledge this study was the largest of unrecognised Ebola virus disease and pauci-symptomatic or asymptomatic Ebola virus-infected contacts so far.

This paper emphasises the public health and clinical care value in identifying contacts with unrecognised Ebola virus disease, who accounted for a substantial population (8·7% of our cohort). Our proof-of-concept study strongly suggests the need for widespread testing of contacts during an Ebola virus disease outbreak so that the prevalence of unrecognised Ebola virus disease can be reduced and post-outbreak surveillance of remaining individuals with unrecognised illness can lead to their identification and linkage to care. The full clinical spectrum of acute viral infections such as SARS-CoV-2 is increasingly recognised to cause post-infectious clinical sequelae.^29^ In conclusion, contacts with unrecognised Ebola virus disease can suffer from post-Ebola virus disease clinical sequelae and are in need of equitable access to care and support services.

## Figures and Tables

**Table 1:** Summary of participant demographics and follow-up

	Survivors (n=991)	Unrecognised Ebola virus disease (n=107)	Pauci-symptomatic or asymptomatic (n=115)	Uninfected (n=2466)	All participants (n=3679)	p value for difference between groups
Sex	··	··	··	··	··	0·62
Female	545 (55%)	66 (62%)	63 (55%)	1374 (56%)	2048 (56%)	··
Male	446 (45%)	41 (38%)	52 (45%)	1092 (44%)	1631 (44%)	··
Median age at enrolment, years (IQR)	29 (19–40)	25 (16–37)	23 (14–34)	23 (14–35)	25 (15–36)	<0·0001
Median log_10_ antibody concentration, EU/mL (IQR)	4·28 (4·1–4·52)	4·19 (3·64–4·45)	3·44 (2·92–3·92)	1·93 (1·69–2·2)	2·2 (1·81–4·01)	*
Median time from Ebola treatment unit or treatment unit discharge to enrolment, days (IQR)	317 (271–366)	NA	NA	NA	NA	NA
Ebola treatment unit or treatment unit discharge date unknown	212 (21%)	NA	NA	NA	NA	NA
Enrolment site	··	··	··	··	··	<0·0001
John F Kennedy Medical Center	618 (62%)	48 (45%)	51 (44%)	1144 (46%)	1861 (51%)	··
C H Rennie Hospital	161 (16%)	28 (26%)	34 (30%)	678 (28%)	901 (25%)	··
Duport Road Clinic	212 (21%)	31 (29%)	30 (26%)	644 (26%)	917 (25%)	··
Completed 12-month follow-up visit	881 (89%)	97 (91%)	101 (88%)	2188 (89%)	3267 (89%)	0·92
Baseline ophthalmic examination	889 (90%)	57 (53%)	39 (34%)	972 (39%)	1957 (53%)	<0·0001
12-month ophthalmic examination	544 (55%)	33 (31%)	24 (21%)	595 (24%)	1196 (33%)	<0·0001

Data are n (%) unless otherwise specified. Categorical variables were compared between groups using χ^2^ tests and age was compared between groups using one-way ANOVA. NA=not applicable.

* See figure 1 for p value.

**Table 2:** Prevalence of selected symptoms and findings on physical examination at baseline and 12 months

	Survivors	Unrecognised Ebola virus disease	Pauci-symtomatic or asymptomatic	Uninfected
**Urinary frequency**				
Baseline visit	143/991 (14%)	3/107 (3%)	5/115 (4%)	83/2466 (3%)
12-month visit	18/881 (2%)	1/97 (1%)	0	21/2188 (1%)
**Headache**				
Baseline visit	474/991 (48%)	54/107 (50%)	37/115 (32%)	879/2466 (36%)
12-month visit	283/881 (32%)	12/97 (12%)	8/101 (8%)	275/2188 (13%)
**Fatigue**				
Baseline visit	180/991 (18%)	12/107 (11%)	7/115 (6%)	152/2466 (6%)
12-month visit	45/881 (5%)	1/97 (1%)	1/101 (1%)	24/2188 (1%)
**Muscle pain**				
Baseline visit	227/991 (23%)	16/107 (15%)	10/115 (9%)	242/2466 (10%)
12-month visit	110/881 (12%)	11/97 (11%)	8/101 (8%)	161/2188 (7%)
**Memory loss**				
Baseline visit	284/991 (29%)	11/107 (10%)	6/115 (5%)	113/2466 (5%)
12-month visit	41/881 (5%)	1/97 (1%)	0	3/2188 (<1%)
**Joint pain**				
Baseline visit	467/991 (47%)	31/107 (29%)	14/115 (12%)	434/2466 (18%)
12-month visit	237/881 (27%)	16/97 (16%)	12/101 (12%)	189/2188 (9%)
**Chest findings**				
Baseline visit	57/991 (6%)	1/107 (1%)	10/115 (9%)	69/2466 (3%)
12-month visit	15/881 (2%)	1/97 (1%)	4/101 (4%)	20/2188 (1%)
**Joint findings**				
Baseline visit	50/991 (5%)	2/107 (2%)	1/115 (1%)	50/2466 (2%)
12-month visit	22/881 (2%)	2/97 (2%)	2/101 (2%)	25/2188 (1%)
**Neurological findings**				
Baseline visit	54/991 (5%)	4/107 (4%)	2/115 (2%)	35/2466 (1%)
12-month visit	14/881 (2%)	1/97 (1%)	0	13/2188 (1%)
**Muscle findings**				
Baseline visit	49/991 (5%)	3/107 (3%)	0	30/2466 (1%)
12-month visit	9/881 (1%)	0	0	9/2188 (<1%)
**Abdominal findings**				
Baseline visit	181/991 (18%)	16/107 (15%)	19/115 (17%)	278/2466 (11%)
12-month visit	111/881 (13%)	12/97 (12%)	10/101 (10%)	205/2188 (9%)
**Uveitis**				
Baseline visit	239/889 (27%)	10/57 (18%)	6/39 (15%)	118/972 (12%)
12-month visit	181/544 (33%)	6/33 (18%)	5/24 (21%)	94/595 (16%)

**Table 3:** Odds ratios (95% CI) for selected symptoms and findings on physical examination

	Survivors *vs* unrecognised Ebola virus disease		Survivors *vs* pauci-symptomatic or asymptomatic		Unrecognised Ebola virus disease *vs* uninfected		Pauci-symptomatic or asymptomatic *vs* uninfected	
	Baseline visit	12-month visit	Baseline visit	12-month visit	Baseline visit	12-month visit	Baseline visit	12-month visit
Urinary frequency	8·14 (2·73–24·29)	2·40 (0·30–19·10)	4·42 (1·79–10·91)	NA	0·73 (0·24–2·17)	0·94 (0·12–7·52)	1·34 (0·53–3·40)	NA
Headache	0·88 (0·58–1·34)	2·99 (1·60–5·59)	1·82 (1·17–2·86)	4·99 (2·37–10·53)	1·78 (1·18–2·69)	0·97 (0·52–1·8)	0·86 (0·55–1·33)	0·58 (0·27–1·23)
Fatigue	1·79 (0·88–3·62)	4·82 (0·66–35·10)	3·21 (1·45–7·11)	5·02 (0·69–36·61)	1·78 (0·87–3·61)	0·93 (0·12–7·01)	0·99 (0·46–2·15)	0·89 (0·12–6·65)
Muscle pain	2·02 (1·14–3·57)	1·40 (0·69–2·84)	3·53 (1·73–7·18)	2·37 (1·15–4·87)	1·53 (0·87–2·69)	1·81 (0·91–3·61)	0·88 (0·43–1·77)	1·07 (0·53–2·17)
Memory loss	4·47 (2·41–8·30)	3·56 (0·49–25·78)	8 (3·47–18·47)	NA	2·06 (1·10–3·84)	7·5 (0·8–70·47)	1·15 (0·50–2·65)	NA
Joint pain	2·13 (1·34–3·39)	1·61 (0·93–2·79)	6·69 (3·70–12·10)	2·24 (1·15–4·36)	1·89 (1·21–2·97)	2·14 (1·23–3·75)	0·60 (0·34–1·09)	1·54 (0·78–3·03)
Chest findings	6·91 (0·95–50·27)	1·91 (0·24–14·92)	0·65 (0·32–1·35)	0·43 (0·14–1·34)	0·31 (0·04–2·28)	1·02 (0·13–7·97)	3·32 (1·63–6·76)	4·55 (1·51–13·75)
Joint findings	3·59 (0·78–16·51)	1·01 (0·22–4·55)	7·10 (0·93–54·00)	0·90 (0·20–4·10)	0·79 (0·17–3·57)	1·73 (0·39–7·65)	0·40 (0·05–3·01)	1·94 (0·44–8·53)
Neurological findings	1·64 (0·62–4·37)	1·21 (0·17–8·4)	3·21 (0·70–14·66)	NA	2·31 (0·87–6·15)	1·71 (0·24–12·13)	1·18 (0·26–5·36)	NA
Muscle findings	3·00 (0·79–11·33)	NA	NA	NA	1·75 (0·45–6·74)	NA	NA	NA
Abdominal findings	1·59 (0·93–2·72)	1·17 (0·58–2·34)	1·12 (0·66–1·91)	1·20 (0·58–2·48)	1·17 (0·70–1·95)	1·10 (0·56–2·18)	1·65 (0·99–2·76)	1·07 (0·53–2·16)
Uveitis	1·72 (0·85–3·48)	2·12 (0·76–5·95)	2·02 (0·82–4·93)	1·77 (0·63–4·92)	1·50 (0·74–3·05)	1·15 (0·41–3·26)	1·29 (0·52–3·16)	1·39 (0·49–3·90)

All odds ratios were estimated using logistic regression models that adjusted for age, sex, and enrolment site (except for the odds ratios for uveitis, which adjusted for age and sex only) and used generalised estimating equations to adjust for relationships between survivors and close contacts. NA=not applicable.

**Table 4:** Incidence of selected symptoms and findings on physical examination, hospitalisation since last follow-up visit, and death within 1 year after enrolment

	Number of cases (cases per 1000 person-years)				Hazard ratio (95% CI)			
	Survivors	Unrecognised Ebola virus disease	Pauci-symptomatic or asymptomatic	Uninfected	Survivors *vs* unrecognised Ebola virus disease	Survivors *vs* pauci-symptomatic or asymptomatic	Unrecognised Ebola virus disease *vs* uninfected	Pauci-symptomatic or asymptomatic *vs* uninfected
Urinary frequency	14 (19·25)	2 (21·40)	2 (20·49)	48 (22·64)	1·09 (0·25–4·76)	0·96 (0·22–4·28)	0·85 (0·21–3·45)	0·96 (0·23–3·99)
Headache	142 (368·24)	15 (340·63)	10 (141·87)	302 (227·71)	0·94 (0·54–1·61)	2·33 (1·29–4·22)	1·50 (0·90–2·52)	0·60 (0·34–1·08)
Fatigue	68 (99·96)	5 (58·84)	6 (62·22)	71 (34·84)	1·56 (0·62–3·91)	1·38 (0·59–3·26)	1·61 (0·65–4·04)	1·82 (0·78–4·25)
Muscle pain	122 (194·88)	15 (190·38)	11 (121·02)	244 (127·42)	0·94 (0·56–1·57)*	1·35 (0·72–2·53)*	1·33 (0·80–2·19)*	0·93 (0·51–1·71)*
Memory loss	50 (85·07)	2 (22·89)	0 (0)	5 (2·38)	3·57 (0·85–14·90)*	NA	9·61 (1·86–49·75)*	NA
Joint pain	112 (277·49)	8 (120·21)	12 (135·34)	209 (119·58)	1·82 (0·94–3·53)	1·64 (0·93–2·91)	1·01 (0·53–1·91)	1·12 (0·64–1·96)
Chest findings	23 (28·37)	6 (63·84)	3 (31·78)	49 (23·11)	0·47 (0·18–1·18)	0·85 (0·26–2·85)	2·62 (1·09–6·29)	1·43 (0·45–4·52)
Joint findings	26 (33·26)	1 (10·72)	2 (19·71)	19 (8·91)	2·1 (0·31–14·4)	1·05 (0·25–4·54)	1·14 (0·17–7·83)	2·27 (0·53–9·70)
Neurological findings	21 (26·19)	0 (0)	0 (0)	9 (4·15)	NA	NA	NA	NA
Muscle findings	13 (16·74)	0 (0)	0 (0)	9 (4·23)	NA	NA	NA	NA
Abdominal findings	81 (120·16)	13 (163·12)	6 (70·87)	187 (97·97)	0·71 (0·39–1·27)	1·56 (0·67–3·67)	1·56 (0·89–2·74)	0·7 (0·30–1·64)
Uveitis	91 (188·90)	3 (99·68)	3 (148·48)	55 (97·33)	NA	NA	0·86 (0·28–2·70)*	2·23 (0·64–7·74)*
Hospitalisation	53 (62·57)	3 (31·31)	4 (39·45)	54 (24·70)	2·16 (0·68–6·79)	1·52 (0·54–4·27)	1·16 (0·36–3·72)	1·64 (0·61–4·42)
Death ≤1 year after enrolment	4 (4·00)	2 (18·80)	1 (8·70)	11 (4·50)	0·21 (0·04–1·21)	0·42 (0·05–3·86)	3·9 (0·79–19·40)	1·94 (0·25–15·30)

Hazard ratios were calculated from Cox proportional hazard models and are adjusted for age, sex, and enrolment site unless noted otherwise. The models used generalised estimating equations to adjust for relationships between survivors and close contacts. Time-to-event for symptoms and hospitalisation was calculated as the number of days from enrolment to the date of the 6-month or 12-month follow-up visit, either the first visit at which the symptom was reported, or, if no symptom was reported, the last visit that took place. Survival time was calculated as the number of days from enrolment to death, follow-up discontinuation date, or the 1-year anniversary of enrolment, whichever occurred first. NA=not applicable.

* Not adjusted for site.

## Research in context

### Evidence before this study

We did a systematic review of studies assessing clinical sequelae among Ebola virus disease survivors, inclusive of individuals with pauci-symptomatic Ebola virus infection and unrecognised Ebola virus disease. We searched PubMed and Web of Science up to Dec 4, 2021, using the search string “ebola AND clinical sequelae NOT vacc* NOT immuniz*”, as well as reference lists and conference reports such as those from the Filovirus Animal Non-Clinical Group Ebola Workshop. We last updated the search on Dec 4, 2021, and used no language restrictions. Although we found no reports specific to individuals with pauci-symptomatic Ebola virus infection and unrecognised Ebola virus disease, we found a large body of evidence that Ebola virus disease survivors experience a wide spectrum of clinical sequelae, ranging from uveitis and memory loss to headache and muscle pain. A substantial proportion of post-Ebola virus disease sequelae persisted for as long as 2·5–4 years.

### Added value of this study

In this longitudinal cohort in Liberia following the 2013–16 Ebola outbreak, we found evidence of post-Ebola virus disease clinical sequelae among contacts with unrecognised Ebola virus disease but not in those with pauci-symptomatic Ebola virus infection. Previous cohort studies of Ebola virus disease survivors were smaller in size than the present study, so even if they had identified a group of contacts with pauci-symptomatic infection and unrecognised Ebola virus disease, they were underpowered, did not use a control group, and were unable to reliably identify the presence or absence of post-Ebola virus disease symptoms and clinical examination findings considered to be clinical sequelae. Our findings were consistent for several symptoms (memory loss, headache, and joint pain), which further supports the concept that post-Ebola virus disease sequelae occur among unrecognised Ebola virus disease contacts. We also found patterns suggesting that more severe acute illness has the potential to cause specific types of post-Ebola virus disease clinical sequelae: memory loss, joint pain, headache, and urinary frequency. These findings extend the evidence for a dose-dependent relationship between acute illness and clinical sequelae, by showing its occurrence across the spectrum of clinical manifestations, particularly among people with unrecognised Ebola virus disease (a group identified as having fewer symptoms than reported Ebola virus disease survivors during acute illness).

### Implications of all the available evidence

This report emphasises the public health and clinical care value in identifying contacts with unrecognised Ebola virus disease, who account for 8·7% of all participants in our cohort. Our proof-of-concept study strongly suggests the need for a natural history study of asymptomatic infection and mild Ebola virus disease illness. Contacts with unrecognised Ebola virus disease can suffer from post-infection clinical sequelae and are in need of equitable access to care and support services.
